# Short Message Service (SMS)-Based Intervention to Improve Treatment Adherence among HIV-Positive Youth in Uganda: Focus Group Findings

**DOI:** 10.1371/journal.pone.0125187

**Published:** 2015-04-16

**Authors:** Yashodhara Rana, Jessica Haberer, Haijing Huang, Andrew Kambugu, Barbara Mukasa, Harsha Thirumurthy, Peter Wabukala, Glenn J. Wagner, Sebastian Linnemayr

**Affiliations:** 1 RAND Health, RAND Corporation, Santa Monica, California, United States of America; 2 Massachusetts General Hospital Center for Global Health, Boston, United States of America; 3 Infectious Diseases Institute, Kampala, Uganda; 4 Mildmay Uganda, Kampala, Uganda; 5 Department of Health Policy and Management, University of North Carolina at Chapel Hill, Chapel Hill, North Carolina, United States of America; David Geffen School of Medicine at UCLA, UNITED STATES

## Abstract

This paper presents one of the first qualitative studies to discuss programmatic barriers to SMS-based interventions for HIV-positive youth and discusses pathways through which youth perceive them to work. We conducted six focus groups with 20 male and 19 female HIV-positive youths in two clinics in Kampala, Uganda. We find that youth commonly use SMS as over 90% of this study’s youths knew how to read, write and send messages and almost three-fourths of them had phones. Youth strongly felt that the success of this intervention hinged on ensuring confidentiality about their HIV-positive status. Key programmatic challenges discussed where restrictions on phone use and phone sharing that could exclude some youth. Participants felt that the intervention would improve their adherence by providing them with needed reminders and social support. Youths’ suggestions about intervention logistics related to content, frequency, timing and two-way messages will be helpful to practitioners in the field.

## Introduction

Young people are at the epicenter of the HIV epidemic. In 2011, some 2,400 youth were infected with HIV every day and youth between the ages of 15–24 accounted for nearly half of all HIV infections [1]. What is equally worrisome is that youth have lower adherence to both HIV antiretroviral therapy (ART) and prophylaxis than adults. A recent review that included more than 50 studies in the area of nonadherence among HIV-positive children and adolescents found that the majority of youth mostly in the developed world showed suboptimal medical adherence [2]. Yet despite a growing literature, there is still no consensus about the best approach to improve adherence and there is a dearth of interventions directed at improving adherence among young people [3].

HIV-positive youth face unique adherence challenges due to their developmental and cognitive stage. Youth have been shown to be particularly prone to forget to take their medicines [4]. Recent research in neuroscience suggests that youth have greater self-regulatory challenges and that they overvalue the present i.e. discount the future at higher rates than adults [5, 6]. Similar findings come from the field of behavioral economics which suggest that attention is a limited resource and that people are often present-biased in their decision-making, showing a marked preference for immediate gratification at the expense of their long term goals [7–9]. Youth may also need greater social support and motivation to counter stigma against HIV status and subsequently improve adherence. Several studies have found that communities where HIV-positive youth experience stigma and discrimination from family and friends are more likely to engage in nonadherence for fear of disclosing their HIV-positive status [10]. Interventions based on targeted pathways that address youths’ limited cognitive capacities and other unique needs may therefore achieve more effectiveness.

Short message services (SMS)-based interventions to improve adherence are low-cost and may be able to address some of the above barriers to adherence faced by adolescents. SMS-based interventions could be particularly suitable for youth who irrespective of their socio-economic status are typically competent users of mobile phones and text messages. Such interventions have been tested across several regions and have resulted in a significant improvement in treatment adherence for patients with different health conditions such as asthma, diabetes and tuberculosis among others [11–13]. With respect to HIV-positive populations, randomized controlled trials have shown that SMS-interventions can increase ART adherence and rates of viral suppression among adults [14, 15]. However, not all SMS-based interventions have demonstrated effectiveness in improving adherence among HIV-positive adults. In a recent randomized controlled trial in Cameroon, HIV-positive adults who received weekly standardized motivational text messages did not have improved adherence outcomes compared to those receiving usual care [16]. In the case of youth, only one small study (n = 25) has assessed the potential of SMS-based interventions to address adherence barriers and found that individualized and interactive SMS daily reminders considerably improved self-reported adherence [17].

**Fig 1:**
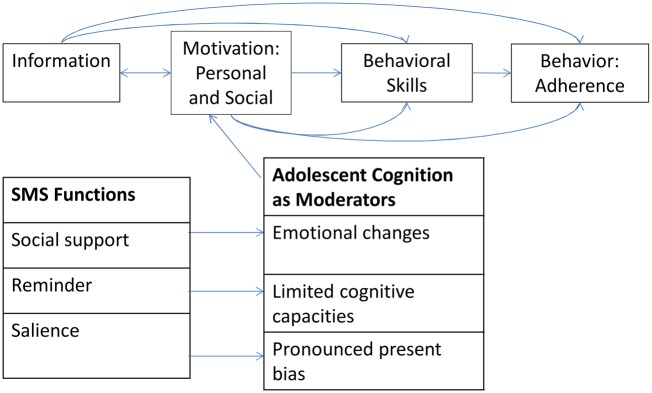
IMB model adapted to RATA intervention.

While SMS-based interventions have shown potential to improve adherence, it is much less clear through which channels (some of which were discussed above) they work. We use the information, motivation and behavioral skills (IMB) model of health as our theoretical framework as it allows us to systematically lay out these channels. The IMB model posits that information is necessary to alter behavior and behavioral skills are needed for more complex tasks, but motivation, both personal and social, is what determines whether an individual acts on that information[18, 19]. As per this model, we hypothesize that SMS messages are likely to positively influence adherence in three important ways (Fig 1). First, at the most basic level, SMS messages may serve as a pure reminder function to address forgetfulness. Second, the message content can provide social support through encouragement (e.g., “stay healthy”; “you can do it”), which we theorize will increase motivation through increased social support and self-efficacy especially during a period of turbulent emotional changes, based on the IMB theory model underlying this analysis. Third, messages can also make the need for and benefits of drug adherence more salient and tangible (e.g., “take your drugs now and be healthy and successful in life”) to counter the pronounced present-biased tendencies of youth.

This qualitative paper reports on HIV-positive youths’ opinions about SMS-based interventions in Uganda. In Uganda, youth aged 10–24 years comprise 33% of the population but account for nearly 50% of the country’s HIV/AIDS cases [20]. The sole qualitative study from Uganda about youth adherence to HIV treatment reported that 71% of the study’s youth had occasionally or frequently missed doses [21]. Ensuring optimal adherence to treatment and preventing the development of drug resistance is especially important in resource-limited settings such as Uganda where second-line therapy is often prohibitively expensive or altogether unavailable.

We present novel findings on youths’ perspectives on the acceptability and feasibility of SMS-based interventions. We also explore pathways through which factors affect medication adherence to develop strategies for best implementing SMS-based interventions. This is one of the first qualitative papers to report youths’ opinions about SMS-based interventions targeted at improving their adherence and so we hope that lessons forwarded here will be helpful to other providers implementing similar programs in developing regions.

## Methods

### Description of Program

The data underlying the study were collected as part of formative work for developing an SMS-based intervention called Reminding Adolescents to Adhere (RATA). A subsequent two-year randomized controlled trial will test the intervention’s ability to prompt HIV- positive youth aged 15–24 with adherence problems in two clinics in Uganda to take their medications on time and offer social support via motivational text messages.

### Sample

In July 2013, we conducted six focus groups (FGs) with 39 HIV-positive youth who were receiving ART from the two clinics Mildmay and IDI in Kampala, Uganda. In each clinic, three focus groups were conducted involving minors of mixed gender, female youth age 18 years and above and male youth age 18 years and above, respectively. We conducted focus groups separately by clinic in order to reflect potentially different clinic environments and/or different types of patients in care at the two sites; by gender to take into account different types of barriers for girls and boys; and, by age as we expected that issues of phone access could be related to school restrictions, and also because we expected minors to be less likely to own their phone. Due to a smaller number of available minor clients in both clinics, we conducted focus groups with minors as mixed gender ones. Each focus group involved the participation of 5–9 clients (median = 6) who had adherence problems defined as having missed at least one medication dose per week on average (self-reported).

Focus group participants were randomly selected. First, the study team used the clinic’s electronic database to determine the age and reported adherence for clients who were expected to visit the clinic during recruitment days (Tuesdays at Mildmay, Wednesdays at IDI). Using the roster, the team developed a registry of eligible participants. Second, a random number generator was used to select eligible participants. Finally, selected participants were approached during their clinic visit and were asked if they would be interested in participating in a focus group. Those who were interested in participating were administered verbal informed consent procedures by the study interviewers. Participants were paid 15,000 Uganda shillings (~$6 USD) to compensate for transportation costs and time. The study protocol and use of oral consent was reviewed and approved by the Institutional Review Board at RAND, Mildmay Uganda, and IDI using the IRB of the Joint Clinical Research Center as well as the Uganda National Science Counsel.

### Interview Structure

Before the start of focus groups, we provided participants a short quantitative survey so that we would have a better understanding of focus group participants. This survey consisted of 13 short items that asked participants to answer questions pertaining to the following domains: a) age; b) adherence challenges; and c) feasibility of intervention which explored the availability of cell phones, familiarity with text messages including being able to read, write and send messages and acceptability of SMS-based interventions to improve adherence among HIV-positive youth. For adherence challenges, participants had to evaluate how confident they felt that they could access and use HIV care along a scale of 0 to 10 with higher scores indicating more confidence while for feasibility related questions they provided yes/no answers.

An interview guide was developed to structure focus groups. The interview guide was guided by the IMB model in that it elicited information about how youth thought SMS-based interventions would function to improve their adherence and the best ways of implementing such interventions. Specifically, participants were requested to reflect upon (1) general reaction to the RATA program (2) cell phone related issues (3) familiarity and comfort with text messages (4) privacy concerns and, (5) strategies for best implementing RATA (frequency, content, and technical/ logistical considerations), as well as potential pitfalls to be avoided and, feasibility of two-way messages. The focus group protocol listed domains and sample questions that were used to guide focus groups.

Focus groups were conducted in the native language of participants, which was typically Luganda, although some of the participants preferred to speak in English, resulting in FGs that often contained a mix of both languages. They were conducted by two bilingual clinic staff, one male and one female both of whom were in their 30’s. Both clinic staff were hired specifically for the purpose of implementing the RATA study, had previous experience conducting focus groups, and also received training in qualitative interviewing methodology (including mock focus groups and role playing) by the study PI (SL). Interviewing was done typically by the person employed at the respective clinic, while the other interviewer took notes and contributed to the discussion as needed. All focus groups were digitally recorded with permission from participants and then translated verbatim into English for data analysis.

### Data Analysis

Data were analyzed for six focus groups. We began the data analysis procedure by reading the six focus group transcripts. During this process, we realized that respondents discussed three themes—perceived benefits of the intervention, perceived challenges of intervention and provided suggestions for best implementing RATA. Under benefits of the program, respondents discussed pathways through which the intervention was perceived to have an impact and so we renamed this theme as pathways mechanisms. Suggestions for implementing RATA included specific feedback regarding content of messages, identity of sender, frequency of messages, use of passwords and feasibility of two-way messages. Consequently, two members developed a codebook of three master codes and five sub-codes outlining inclusion and exclusion criteria for each code. We used Atlas.ti to systematically code connecting blocks of text pertaining to the codes and sub-codes. Once coding was complete, we extracted all text associated with each code and sub-code and examined the text to identify common themes. For new themes, we further created sub-codes, added definitions to the codebook and used Atlas.ti to mark similar text belonging to the new codes. Through discussions, YR and PW developed the codebook. YR coded all six transcripts while PW checked coded outputs.

## Results

### Sample Characteristics and Perceived Barriers to Treatment

Table 1 provides information about participant characteristics, perceived barriers to treatment and feasibility of the intervention.

**Table 1:** Participant Characteristics.

	Mean +- S.D.	Range
**Total Number of participants**	39.0	
**Background**
Age	19.5+-2.9	14.0–24.0
**Treatment related**
Take medication as directed	8.4+/-1.7	4.0–10.0
Understand instructions received at clinic	8.7 +/- 1.9	3.0–10.0
Have all information needed to pursue treatment	7.8 +/- 2.7	2.0–10.0
Return for scheduled appointments	8.9+/-2.0	4.0–10.0
Disclosure about HIV status	4.7 +/- 3.2	0.0–10.0
**SMS-based interventions**
Own cellphone	72.0%	
Share phones	41.0%	
Family (mother, siblings)	69.0%	
Partner	19.0%	
Friends	12.0%	
Knows how to write a text	92.0%	
Knows how to read a text	97.0%	
Uses cell phone to send texts	92.0%	
Intervention anticipated to be helpful in improving adherence	97.0%	

Of the 39 focus group participants, 11 participants (6 male and 5 female) were in the minor focus groups, and 14 young males and 14 young females were in the gender-specific non-minor focus groups of youth 18 years or older. The average age was 19.5 years (S.D = 2.87). On a scale of 1–10 with 10 indicating the highest level of confidence, more than 50% of the study sample reported a score of 10 when they were asked whether they could understand instructions provided at clinics and whether they could attend scheduled appointments on time. 56% of youth also reported a score of 10 when they were asked if they had all the information needed to pursue their treatment. However, only one-third of youth were fully confident (score of 10) that they could take medication as directed and only 10% (score of 10) talked about not feeling ashamed in disclosing their HIV positive status.

### Feasibility of the Intervention

Ninety percent of the sample reported knowing how to write, read and send texts, and 72% reported owning a cell phone. Nearly half of sample participants (41%) reported sharing phones with others. Youth were most likely to share phones with family members such as mothers, grandmothers and older siblings. Few youth also reported sharing phones with their partners and friends. In general, almost all participants (97%) felt that the RATA intervention would help them improve their adherence.

### Programmatic Challenges and Suggestions

#### Perceived Challenges to the Intervention

Participants identified four main challenges to the implementation of RATA. First, not all youth have access to mobile phones. A typical concern expressed by one older male youth, “I’m seeing me benefiting but probably not so many others…will everyone have access to a mobile phone?” This quote represents the worry that some participants had about youth without phones being unduly excluded from the study.

Second, sharing phones would be problematic for those youth who have not disclosed their status to those with whom they share phones. In the words of one older female youth, “You can be attending [the HIV clinic] and none of your family members knows that you are on treatment. So I may fear to give out my father’s phone number because you might call him and tell him that I’m your client here at [the HIV clinic] and I worry for what might happen after that.” Another complication may arise when owners of the mobile phones forget to inform youth about their messages or get tired receiving messages. The following quote elaborates one possible scenario where parents can forget, “The challenge is that most of us on treatment do not have phones of our own in that if you send a message to parents many of them do not care; they will not deliver the messages because they are busy. The parent may say, he will remind himself” (minor youth).

Third, some youth reported facing restrictions on their phone use. In general, both older and younger youth discussed being prohibited from bringing to and using mobile phones at schools and after a certain hour at home for young and female youths. The large context of the following quote fits in with other similar quotes on this theme, “If they get you with a phone they suspend you or expel you which will bring more trouble to you” (older female youth). With respect to restrictions at home, youth reasoned that their parents restricted their phone use because they would spoil them by “making them get bad influence from wrong people,” since they feared that youth had not reached of an age to “distinguish between good and bad.” According to one older male youth, “They don’t restrict me, because I’m a boy ….unlike the girls who will not be allowed to have phones, if they have them it’s by force and they make sure they hide them from the parents.” To overcome restrictions at schools, youth noted that they or their next of kin would have to discuss their problem to solicit the support of the school which one older female youth did not want because she was not ready to disclose her status. This limitation could severely affect the impact of the proposed intervention since youth felt that the messages would be most effective in helping adherence if they came right before youth are expecting to take medications.

Fourth, youth unanimously voiced concerns about accidental disclosure of HIV status because others may knowingly or unknowingly read their messages when their phone is not with them. Youth expressed a range of opinions about how disclosure might take place:

“One may ask you for your phone and when you give it to them they may read your messages and begin spreading rumors about you.”

(older male youth)

“Some of us don’t have power at home especially those in villages so when you take your phone to charge, someone might read your messages.”

(older female youth)

“I myself might be very busy and I leave my phone there and somebody comes and picks it. He does not know about my status and we are like at work, he opens my phone and sees [name of clinic], eeh, what is that, what do they do, HIV testing and treatment, ooh, so this man is dead!”

(older male youth)

“When you send an SMS, it becomes an exposure to many; one the company sending the SMS, secondly those tapped communications systems, so it becomes an exposure.”

(older male youth)

In the event of breach of confidentiality, youth feared facing stigma and isolation.

In addition to the above-mentioned challenges, youth also raised concerns about the sustainability of the program. Some youth mentioned that over time youth might get used to and bored by messages and begin to ignore them. A prototypical quote included, “we may get used and bored by the messages thinking that they are always about reminding” (older male youth). A few youth were worried that the program would create dependency among youth.

“We don’ know how consistent it will be, you could be starting it 2013 and come 2016 there is a disruption somewhere and it stops suddenly. We are not sure whether it’s going to be consistent because the thing [ARVs] that we are on is for a lifetime experience.”

(older male youth)

The issue raised in this quote is similar to the concern raised in other quotes in that youth are worried that the program might make their minds “dormant” and dependent on text messages and it is not clear if the program can be maintained forever.

#### Suggestions for Message Content

Participants in five out of the six focus groups argued for coded messages that would help maintain confidentiality. Overall, there was a general consensus that it would be best if the messages did not contain the words ‘drugs’ or ‘pills’ so as not to compromise participants’ HIV status. Participants indicated that it would be better to have pre-agreed-on, coded messages. The following quote elaborates one participant’s desire to keep the real meaning of the messages anonymous so that even if others accessed his phone they would not know about his HIV status, “the message I think you should come up with are more like a code which will be used and it will be told to all the youth who come to the clinic here like when you see this code in your message, just know that it is reminding you of this activity.”

Participants collectively suggested keeping the content of the message straight-forward and simple so that it would not raise any suspicions. Some suggestions forwarded were, “hi, how are you doing?” “is it time,” “swallow,” and depending on the time of the day “have you had your tea/ dinner?” Some participants also suggested sending inspirational messages such as “your future is better than today” or issues that bring youth together and are common across age groups such as “music,” or “sports.” In addition, a few participants recommended changing the content of the message so that they don’t get tired and bored reading the same messages. Suggestions included sending information on latest health updates, family planning and other lifestyle related issues and switching between reminder and motivational messages, “if today you send a message to remind me then the other day you should send me something like to encourage, motivate or make me happy” (older female youth).

Also, few older female youth who shared phones with their families suggested that it would be better to keep the content of the messages straightforward so that their caretakers know that the message is for them. An example of such concern expressed is as follows, “Unlike if you wrote a straightforward message please such as *don’t forget to take your medication* they will be able to understand who it is for. I know for us we can understand the RATA thing but our caretakers won’t so I would suggest that we use the right words like go straight to the point” (older female youth).

#### Suggestions for Identity of the Sender

Most focus group participants preferred not disclosing the clinic as the sender of the message primarily because of the stigma that participants feared that they would experience if others in their social circle knew about their HIV-positive status. According to participants, “They know it is somewhere for treatment so when they see the message they will know that you are asking me how I’m doing with my drugs,” and, “I may be having a girlfriend who is not aware of my HIV status; if you send a message from the clinic this person may ask you what it means and you may not want to tell everyone.” Youth, however, recommended specifying the identity of the person so they wouldn’t ignore their messages. One older male youth expressed his fears, “For me I support the name [of the clinic] because sometimes you are busy and a number comes up and you wonder where it is coming from but if the word friend comes you just know what it’s about.” Some of the minor youth participants had disclosed their status to their loved ones and so they had no problems identifying the sender of messages as the clinic from where they are receiving medication. Other participants recommended allowing recipients to personalize the identity of the sender on their own, similar to ideas forwarded for content of messages.

#### Suggestions for Frequency of the Messages

There was no consensus as to the number of times messages should be sent in a week. Some focus group participants preferred receiving messages every day as long as the sender is able to send since it would act as a reminder. Others mentioned wanting to receive messages at least once, twice or three times a week especially during the weekends when they face more distractions and are likely to forget things or on Mondays since it is the first day of the week.

Most participants felt that the best time to send these messages would be right before or at the time youth are likely to be taking their medications. The following quote elaborates youths’ preference for receiving messages at times when they are likely to forgot to take their pills, “For me I take my pills at 9pm so if I get the SMS at about 7pm when I’m on my way home this reminder remains in my mind such that as soon as I get home I take my medicine” (older male youth). Other good times to send messages included during the mornings so that youth can organize their day including taking their pills and during times when youths’ phones are likely to be on. One focus group of minor youth specifically suggested receiving messages in the late evenings so that they could access messages:

“I suggest that because as for my grandmother’s phone that is the only time it’s on because the other time she is always at church.”

(minor youth)

“When a message comes they tell me that there is a message so I can check it unlike in the morning when I’m not at home but at school, but 7 or 8pm is good for me.”

(minor youth)

Respondents also provided suggestions regarding what times would be unsuitable to receive messages. Recommendations included hours when working youth would be on duty and school going youth would be in classes, midnight for some youth because they would be sleeping and, at times when caretakers are unable to relate messages back to youth who share phones with them.

#### Suggestions for Password Protection

We solicited youths’ opinions about using passwords to access text messages since passwords are one way to address privacy concerns. While older youth in focus groups thought that it was a good idea to use passwords, they raised three important concerns. First, not all phones have the facility to install passwords. Second, the presence of a password can raise suspicions especially for youth in relationships and so either they might be requested to remove passwords or share it with their partner. An older male youth talked about his past experience in which he was asked to remove passwords because, “Someone could read them [messages] on account that they wanted to know the person they were going to date.” Instead of passwords, one older male youth suggested using a simple yet coded message that would not raise any suspicions as elaborated by the following quote:

“Things that are put in the open don’t really quiz people’s mind. If you just come up with a message and put it in a way only for me to understand for example “how was your night?” from a friend. You can just connect it to the network people and say these people just want me to keep on their network.”

(older male youth)

The third challenge raised in one focus group was that some people are skilled at removing passwords and so having this facility does not always guarantee security.

#### Suggestions for Two-way Messages

Focus group participants liked the option of being able to reply to messages. They felt that this would allow them to keep in touch with their providers especially when they are feeling unwell. As one minor youth put it, “Sometimes they give us the [appointment], the time to come back here, they give us like one month or half a month like that, so it can be better if we receive those calls. Because before we come back, one can get a serious problem and he or she cannot access the clinic very fast, so if she/he calls, he can be helped in the other way.” For the two-way system to work, participants noted that the content of the message should be coded in such a way that requires them to reply back and ensures them that message has come from the intended sender. However, the common challenge that participants identified was the cost of reply messages. Participants would love to call or text back but they felt that in many cases they could not afford the airtime. A typical quote included, “There may be instances when I cannot reply because I do not have airtime but otherwise I would have loved to write back” (older male youth). Other concerns included delay in replying back because participant was busy, phone was switched off, message reached the intended sender or not and, whether phone has the facility to reply back.

#### Other Suggestions

In addition to the above-mentioned suggestions, respondents also forwarded a range of other recommendations. First, there can be cases where youth use multiple SIM cards and so program administrators should inquire which number should be used for text messaging. Second, in the case of phone sharing, the program should counsel primary care givers or others with whom the youth shares phone so that they know what the project is about because one female older youth complained that “some caretakers are not easy.” Third, youth felt that program administrators should offer the program to youth who have newly started on medication to help them develop a routine. The following quote elaborates why youth who have newly started treatment are more likely to benefit from the program, “You see if a person has just began taking drugs, he or she will take like for one week and he will say after all I don’t have anybody to say, you, come and take the drugs, come and do this. He or she will just leave the drugs systematically from there” (female older youth). Fourth, focus groups of older male and female youth belonging to the same clinic also suggested using other mediums to provide reminders such as voicemail, facebook and alarms:

“Unlike that aspect of the phone where some parents restrict their children like xxx has said he has just got his first phone mobile phone in senior six which means way back when he was in primary, secondary O- level he could not have the phone before not until he had finished A-level, but that person would have been introduced to face book may be in senior one.”

(older male youth)

“Most people tend to ignore messages so I would rather you call the person other than sending messages.”

(older female youth)

“If it’s for reminding me to take my medications it should be an alarm. If you have something else to communicate then send a message.”

(older female youth)

### Pathway Mechanisms

Participants in all six focus groups perceived RATA as being beneficial to youth and their health. The two major benefits that participants outlined in discussions were that the intervention would provide youth with much-needed reminders and social support. Youth felt that messages would be most helpful to those youth who tend to forget or who are so busy that taking medication slips their minds. An illustrative quote included, “There are those who are just forgetful like someone missed to take the drugs and wakes up one morning, I missed to take my drugs, maybe I will take today and then that day, he forgets again and you wonder what is wrong. So I think it is very good to send the message because it will remind that person” (male older youth).

In addition, text messages could also provide youth with “another hand looking out to help those who are helpless.” Participants reasoned that the provision of social support would make them feel that there is someone caring for them which in turn would function to encourage and motivate them to adhere to their treatment. One male older youth explained, “Some of our parents do not care at all, it’s up to you to make sure that you take your medication. When the clinic calls me I feel loved, cared for and this encourages me.” Another female older youth added, “Me, I would feel like yeah, there is someone caring about me and I will do whatever it takes at least not to let that person down.

## Discussion

The purpose of the focus group discussions presented here was to evaluate feasibility and acceptability of SMS messages among HIV-positive youth, to elicit suggestions for the implementation of specific SMS features, and to infer the potential pathways through which the intervention is perceived to work. The results show that youth living with HIV commonly use and are familiar with SMS. Over 90% of the youth in this study knew how to read, write and send text messages and almost three-fourths of them had their own phones. In addition, 97% of the sample anticipated RATA would be helpful towards improving their treatment adherence. When viewed in the context of the widespread use of mobile phones, SMS-based interventions have promising potential to address adherence barriers among HIV-positive youth in a setting such as Uganda.

Regarding the implementation of our SMS intervention, the most important recommendation forwarded by youth was to maintain confidentiality about their HIV-positive status and their being in HIV care. Past studies have found that HIV-related stigma impact peoples’ abilities to adhere to treatment by lowering their social support and ability to cope [22]. This study’s finding is in line with findings from other studies that have noted that participants using text messages are worried about stigma, privacy and confidentiality since text messages can leave a trail of evidence [23]. Like elsewhere, in Uganda privacy is a critical issue since HIV stigma still exists in many communities leading to negative attitudes and maltreatment against youth living with HIV/AIDS and their families [24].

Due to stigma, participants had reservations about the content of text messages and identity of the sender. Participants suggested having simple coded messages without the words ‘drugs’ or ‘pills’ that would function to remind youth to take their medications without raising suspicions. However, youth who shared phones with other family members feared that others may discard coded messages not realizing they are meant for the youth. Youth also recommended allowing recipients to personalize the identity of the sender or agreeing to a name for the sender of messages that only group members would know. Participants were not optimistic about the functionality of using passwords because for youth especially in relationships the presence of a password could indicate infidelity in the relationship. Since past studies that incorporated participant feedback to generate message content have had greater effectiveness [25], future program implementers and policymakers should pay attention to these suggestions.

Youth also discussed two key programmatic challenges. The first limitation includes restrictions on using mobile phones which is mostly faced by younger youth at schools. The second group includes some youth who might not be allowed to use the phone at home after a certain time at night. Another challenge includes phone sharing since text messages can disclose the HIV-status of youth to those with whom they share phones. Both challenges will require program administrators to work closely with key stakeholders to develop interventions addressing these concerns.

The findings also shed some light on our hypotheses about the potential pathways through which SMS-based interventions may affect adherence. From the surveys and focus groups administered, we find that the majority of HIV-positive youth were confident that they had adequate knowledge about their treatment yet program participants were suffering from poor adherence. Hence, information in itself does not seem to be sufficient to induce optimal adherence. As depicted in Fig 1, youth-specific cognitive traits may have a moderating effect on motivation and SMS messages may have the potential to positively influence motivation and impact behavioral skills. In focus groups discussions, youth noted that receiving messages could provide them with much needed social support that they believe would function to motivate and encourage them to look after their health. This feature of support is especially critical in the stigmatized environments that most of these youth live in and that prevents them in many cases from disclosing their status to even their family and close friends.

Along with social support, SMS-based interventions can function as reminders to address forgetfulness, either by providing just-in-time information that a pill dose is due (if the messages are sent at high frequency) or more generally of the importance of taking one’s drugs (when sent less frequently, as in our study). In programmatic suggestions, participants therefore voiced that messages would be most effective if they were sent around the time participants were expected to take medications. This finding is in line with similar findings from a review study that found that a greater treatment effect was observed in studies in which messages were sent at the time of a dosage [25]. Increased salience may come about if participants are reminded of the importance of taking their pills regularly because of the SMS messages. However, while participants seemed to largely favor high-frequency messages to battle forgetfulness, Pop-Eleches et al. (2013) tested weekly- versus daily messages (which at least for those on a one-pill-per-day regimen is close to just-in-time information about pill-taking) and found weekly messages to be more impactful [14]. This highlights the possibility that forgetfulness may actually mask other barriers to optimal pill-taking, such as stigma or lack of social support that for example could make it likely that a youth is unwilling to think about pill-taking, in particular when in company of other youths s/he has not disclosed to. Such more structural forces underlying reported forgetfulness are likely targeted also by less frequent messages that are less likely to suffer from potential fatigue of receiving messages with high frequency.

The group dynamics in the focus groups allowed us to solicit disparate views on the topics of interest. This was also evidenced in the often lively discussions that developed among focus group participants and that highlighted the often differing opinions on particular topic. However, this study has some limitations that should be considered when interpreting results. This study’s findings may not be applicable to HIV-positive youth with sub-optimal adherence in other countries. We could have obtained richer narratives had we further classified focus groups on other factors such as location (rural versus urban) and socio-economic status. Additionally, the definition of feasibility used in this paper relates to the attitudes of patients only. At the time of writing of this paper, we were not yet at a stage to discuss technical feasibility and logistical considerations such as how to send bulk messages or whether or not to reimburse participants in the two-way group for airtime. Many of these issues have since been carefully addressed and will be presented in a paper describing the intervention phase of the RATA study.

## Conclusion

In summary, this formative study demonstrates that SMS-based interventions are likely feasible and have the potential to be acceptable as a means to improve ART adherence among youth in HIV care if issues related to confidentiality and privacy are appropriately taken into account in the program design. A key contribution of our study is that it provides insights from youth that can contribute to our understanding of the pathways through which such SMS-based interventions may affect medication adherence. We will further investigate these pathways as part of the Phase 2 randomized controlled trial of RATA.

## Supporting Information

S1 Appendix Focus Group Guide. (DOCX)
