# Supplementary material for: Short Message Service (SMS)-Based Intervention to Improve Treatment Adherence among HIV-Positive Youth in Uganda: Focus Group Findings
Source: PLoS One. 2015 Apr 16;10(4):e0125187. doi: 10.1371/journal.pone.0125187 (PMC4400100; doi:10.1371/journal.pone.0125187)
Supplement: S1 Appendix — (DOCX) [file pone.0125187.s001.docx]

**Focus Group Guide**

I would like to present a program to you that we think may help young people in HIV care find it easier to take their medication. It is called RATA (Reminding Adolescents to Adhere). RATA will send text messages with words of encouragement to clients in HIV care such as yourself on a regular basis to help remember and feel motivated to take medications.

**General reaction**

- *In general, what do you think about such a program?*
- *Would XX clients be happy to participate in such a program and receive text messages, or are there any concerns you would have?*

**Cell phone related issues**

- *Would you say that most people your age have cell phones, or is it rather rare?*
- *Would you say that it is typical to share a phone with someone else (such as a family member)?*
- *Are there restrictions to the use of your phone by: your school, parent/guardian, and employer (please explain to what extent and will this prevent you from participating in this study)?*
- *Have you heard of people using several phones at once, or several SIM cards in one phone?*

**Text messaging issues**

- *Do most people your age know how to use cell phones, and in particular how to send and receive text messages? Have you ever sent an SMS?*
- *Do you think it is acceptable for clients such as yourself to receive health-related texts?*
- *How would you feel about getting text messages from the clinic?*

**Privacy issues**

- *Is there concern that messages may be read by someone else but the intended recipient?*
- *Can you tell us about an instance where someone read your texts that made you uncomfortable?*
- *What advice would you have for someone like you on how to protect the privacy of their text messages?*
- *How do we know that the client is the one responding to the message; for those that may require replies?*

**Program design of RATA**

Now we would like to get your feedback on more specific program issues.

- *Frequency of messages: a) How often would you like to get such messages?*
- *Timing of messages: a) what time of the day is best in your opinion to receive such a health-related message? Morning, afternoon, evening? Why do you think so?*
- *Content of messages: Now, we want to hear from you what the text of a message could look like? Remember that the text messages must be short – only 5-10 words.*
- *Passwords:* *Did you know that one can send a sms with a password? Let me hear your views.*
- *Two way messages: One feature of the program that we have not talked about yet is that some of the participants will have the possibility to respond to such a text message and tell a nurse or health care provider how they are doing, whether they need help, etc. What do you think about this idea? Would you think that following up with another text message or by phone call is better?*

This is the end of the group discussion. Thank you and please remember not to share any information about participants in this focus group or give their identity away.
